# Identification of *Mycoplasma* species and related organisms from ruminants in England and Wales during 2005–2019

**DOI:** 10.1186/s12917-021-03037-y

**Published:** 2021-10-12

**Authors:** Alannah S. Deeney, Rachael Collins, Anne M. Ridley

**Affiliations:** 1 Mycoplasma Team, Department of Bacteriology, Animal and Plant Health Agency, Weybridge, Surrey, UK; 2 Animal and Plant Health Agency Veterinary Investigation Centre, Starcross, Exeter, UK

**Keywords:** Mollicutes, Mycoplasma, Ruminant, PCR-DGGE, Pneumonia, Arthritis, Mastitis

## Abstract

**Background:**

*Mycoplasma* species have been associated with economically important diseases affecting ruminants worldwide and include contagious bovine pleuropneumonia (CBPP), contagious caprine pleuropneumonia (CCPP) and contagious agalactia, listed by the World Organisation for Animal Health (OIE). The Mycoplasma Team at the Animal and Plant Health Agency provides an identification service for *Mycoplasma* and *Ureaplasma* species of veterinary importance to the United Kingdom (UK), supporting the detection of new and emerging pathogens, as well as contributing to the surveillance of endemic, and the OIE listed diseases exotic to the UK. *Mycoplasma* and other *Mollicutes* species were identified from diagnostic samples from farmed ruminants in England and Wales using a combination of culture and 16S rRNA gene-based PCR-denaturing gradient gel electrophoresis, submitted between 2005 and 2019.

**Results:**

A total of 5578 mollicutes identifications, which include mycoplasmas and the related acholeoplasmas and ureaplasmas, were made from farmed ruminant animals during the study period. Throughout the study period, the pathogen *Mycoplasma bovis* was consistently the most frequently identified species, accounting for 1411 (32%) of 4447 molecular identifications in cattle, primarily detected in the lungs of pneumonic calves, followed by joints and milk of cattle showing signs of arthritis and mastitis, respectively. *M. bovirhinis*, *M. alkalescens*, *M. dispar*, *M. arginini* and *Ureaplasma diversum*, were also common. Mixed species, principally *M. bovis* with *M. alkalescens, M. arginini *or* M. bovirhinis* were also prevalent, particularly from respiratory samples. The non-cultivable blood-borne haemoplasmas *Candidatus* ‘Mycoplasma haemobos’ and *Mycoplasma wenyonii* were identified from cattle, with the latter species most often associated with milk-drop. *M. ovipneumoniae* was the predominant species identified from sheep and goats experiencing respiratory disease, while *M. conjunctivae* preponderated in ocular samples. The UK remains free of the ruminant mycoplasmas listed by OIE.

**Conclusions:**

The continued high prevalence of *M. bovis* identifications confirms its ongoing dominance and importance as a significant pathogen of cattle in England and Wales, particularly in association with respiratory disease. *M. ovipneumoniae* has seen a general increase in prevalence in recent years, notably in coughing lambs and should therefore be considered as a primary differential diagnosis of respiratory disease in small ruminants.

## Background

The class *Mollicutes* comprises over 200 species of bacteria. These organisms lack a cell-wall containing peptidoglycan; instead, they are bound only by a trilaminar membrane which resembles a typical plasma membrane [1, 2]. Of the five genera within *Mollicutes*, *Mycoplasma* and *Ureaplasma* are associated with disease in ruminant animals and infection by some species can have severe implications for animal welfare and the economic health of farming and livestock production industries worldwide. Contagious bovine pleuropneumonia (CBPP), contagious caprine pleuropneumonia (CCPP) and contagious agalactia are listed by the World Organisation for Animal Health (OIE), owing to their transmissibility and severe welfare and economic consequences; these important diseases remain exotic to the UK.

Other diseases associated with *Mycoplasma* and *Ureaplasma* species endemic to countries around the world are not OIE listed, but nonetheless cause significant production losses, with the infecting organism often difficult to eliminate once established in affected herds and flocks. Most notably is *Mycoplasma bovis*, a cause of pneumonia, arthritis and mastitis in cattle worldwide [3]. The recent introduction of *M. bovis* disease to New Zealand prompted the establishment of drastic eradication measures [4]. Other species implicated in diseases of varying severity and clinical signs include *Mycoplasma dispar*, *Mycoplasma alkalescens, Mycoplasma canadense*, *Mycoplasma californicum*, *Mycoplasma bovigenitalium*, *Mycoplasma bovoculi, Mycoplasma leachii*, *Mycoplasma wenyonii, Mycoplasma canis and Ureaplasma diversum* [5–7], although not all are believed to be primary pathogens. In sheep and goats, *Mycoplasma ovipneumoniae,* an often overlooked cause of respiratory disease and *Mycoplasma conjunctivae*, a major cause of infectious keratoconjunctivitis (IKC) in domestic and wild ovine and caprine species, are important pathogens endemic to the UK [5].

In recent years a combination of microbiological culture and polymerase chain reaction (PCR) have generally replaced traditional serum inhibition tests. Immunohistochemistry of diseased tissues and fluorescence in situ hybridisation are also used to complement microbiology or used alongside clinical signs for disease diagnosis. However, as over 40 different *Mycoplasma* species have been recovered from ruminants, application of specific PCRs can be very challenging and time consuming for identifying multiple species. Consequently, in many diagnostic laboratory settings there is focus on a disease syndrome, or the major pathogens, using simplex or, increasingly, multiplex PCR formats.

The Mycoplasma Team of the Animal and Plant Health Agency (APHA) provides an identification service from submitted clinical samples, for the Department for Environment, Food and Rural Affairs (Defra), providing diagnosis and surveillance of new and emerging *Mycoplasma* and *Ureaplasma* species, as well as those causing endemic disease within the UK livestock industry. This also enables surveillance for species that are exotic to the UK. Identifications of *Mollicutes* species were made by traditional serological tests, such as growth inhibition and fluorescent antibody tests combined with selected species-specific PCRs [8]. However, since 2005, this has largely been replaced at APHA by a culture and PCR-denaturing gradient gel electrophoresis (DGGE) approach for identification of these organisms from a variety of clinical diagnostic sample types including lung and mammary tissue, respiratory lavage, joint aspirate, milk, blood, semen, cerebrospinal tissues and various swab samples. The method, which detects sequence differences within known variable regions of the 16S rRNA gene, can identify different species in any sample type, including mixed infections and facilitates determination of emerging or novel mollicutes [9, 10].

*Mollicutes* identifications from ruminant samples submitted to APHA laboratories in England and Wales to assist disease diagnosis during 2005 to 2019 were analysed and are described.

## Results

### Sample submission trends

**Fig. 1:**
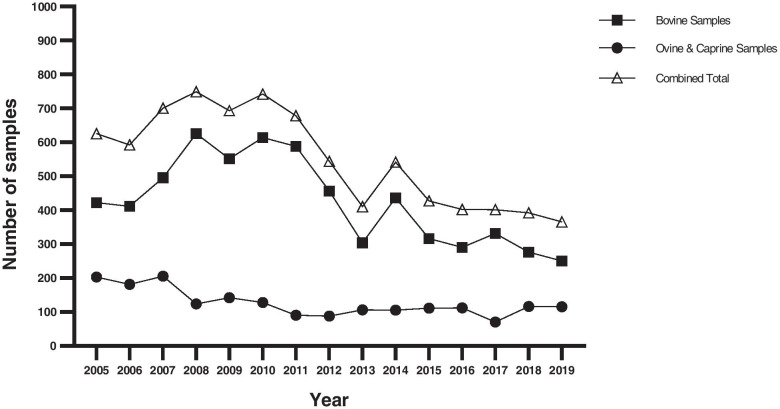
Samples submitted from bovine, ovine and caprine species and examined by molecular DNA tests by the APHA Mycoplasma Team during 2005–2019

The overall number of ruminant samples from England and Wales submitted to APHA for mycoplasma identification increased from 2006, reaching a peak between 2008 and 2010 (Fig. 1), with an average of 420 between 2013 and 2019. The number of bovine samples received consistently exceeded those of small ruminants and peaked at 625 in 2008, with an average of 315 samples annually between 2013 and 2019. Submitted ovine and caprine diagnostic samples peaked in 2007 at 205, with an average of 105 samples over the last six years (Fig. 1).

### Identified species

Overall, 5578 *Mollicutes* species identifications were made from ruminant samples sent as part of diagnostic investigations, of which 4447 were from cattle (Table 1), with the remaining 1131 from sheep and goats (Table 2).

**Table 1:** *Mollicutes* species identified from bovine clinical diagnostic samples by molecular DNA tests at APHA Weybridge during 2005–2019

***Mollicutes species***	Year															Total	%
	2005	2006	2007	2008	2009	2010	2011	2012	2013	2014	2015	2016	2017	2018	2019		
***M. alkalescens***	43	65	46	53	69	34	30	27	31	61	45	41	42	59	31	677	15.22%
***M. arginini***	8	22	20	12	11	8	20	13	20	23	11	15	43	37	23	286	6.43%
***M. bovigenitalium***	8	11	2	12	4	1	1	7	2	0	1	0	1	0	0	50	1.12%
***M. bovirhinis***	115	120	147	78	75	50	40	29	25	30	19	34	48	46	50	906	20.37%
***M. bovis***	113	126	156	82	63	48	67	66	73	123	80	87	128	110	89	1411	31.73%
***M. bovoculi***	0	0	0	0	2	0	1	3	0	1	0	0	0	2	1	10	0.22%
***M. californicum***	0	0	0	0	0	0	0	0	0	0	0	0	1	0	0	1	0.02%
***M. canis***	4	2	1	8	10	7	17	25	23	34	18	10	13	3	0	175	3.94%
***M. canadense***	0	14	0	20	14	3	7	1	5	6	2	6	8	4	1	91	2.05%
***M. dispar***	1	3	3	12	9	11	29	35	47	47	26	56	53	91	55	478	10.75%
***M. equirhinis***	0	0	1	0	0	0	0	0	0	0	0	0	0	0	0	1	0.02%
***M. fermentans***	0	0	0	0	0	0	0	0	0	0	0	0	0	1	0	1	0.02%
***Ca.*** **M.** ***haemobos***	0	0	0	0	0	0	6	0	0	0	0	0	1	1	0	8	0.18%
***M. wenyonii***	0	2	2	21	7	10	12	31	8	33	22	10	8	9	2	177	3.98%
***U. diversum***	0	0	0	0	0	0	1	10	22	10	7	19	14	32	19	134	3.01%
***A. equifetale***	0	0	1	0	0	0	0	0	0	0	0	0	0	0	0	1	0.02%
***A. granularum***	0	0	2	0	0	0	0	1	0	0	0	0	0	0	0	3	0.07%
***A. laidlawii***	4	3	10	8	5	0	0	0	0	0	0	3	1	0	2	36	0.81%
***A. oculi***	0	0	0	0	0	0	0	0	0	0	0	0	0	0	1	1	0.02%
**Total**	296	368	391	306	269	172	231	248	256	368	231	281	361	395	274	4447	

The frequency of identification of each *Mycoplasma, Ureaplasma* and *Acholeplasma* species per year is shown, and the percentage proportion of each species identified

**Table 2:** *Mollicutes* species identified from ovine and caprine clinical diagnostic samples by molecular DNA tests at APHA Weybridge during 2005–2019

***Mollcutes*** species	Year															Total	%
	2005	2006	2007	2008	2009	2010	2011	2012	2013	2014	2015	2016	2017	2018	2019		
***M. arginini***	47	43	38	19	20	11	17	12	16	13	7	17	5	31	27	323	28.56%
***M. bovis***	0	0	0	0	0	0	1	0	0	1	0	0	0	0	0	2	0.18%
***M. conjunctivae***	7	18	24	8	14	5	7	7	14	4	11	12	14	16	26	187	16.53%
***M. ovipneumoniae***	87	97	92	19	47	15	24	24	22	19	34	28	21	61	26	616	54.47%
***M. fermentans***	0	0	0	0	1	0	0	0	0	0	0	0	0	0	0	1	0.09%
***M. ovis***	0	0	0	0	0	0	0	0	0	0	0	0	1	0	0	1	0.09%
***A. laidlawii***	1	0	0	0	0	0	0	0	0	0	0	0	0	0	0	1	0.09%
**Total**	142	158	154	46	82	31	49	43	52	37	52	57	41	108	79	1131	

The frequency of identification of each *Mycoplasma* and *Acholeplasma *species per year is shown, and the percentage proportion of each species identified

*M. bovis* was most commonly identified, accounting for 32% of the species identified over this period. *M. bovirhinis* (20%), *M. alkalescens* (15%), *M. dispar* (11%)*, M. arginini* (6%), *M. wenyonii* (4%), *M. canis* (4%), *U. diversum* (3%), *M. canadense* (2%), and *M. bovigentialium* (1%) were also identified (Table 1)*.* The majority of mycoplasmas identified originated from lower respiratory tract samples, with *M. bovis* being most common in bovine lungs, accounting for 1000 of the 1277 total *M. bovis* identifications in relation to anatomical site of isolation (78.3%; Table 3). *M. bovis* was also the most common single species identified from milk samples, but it was also found mixed with *M. canadense, M. alkalescens* or *M. bovirhinis. M. alkalescens, M. bovirhinis* and *M. canadense* were also observed as sole species. *M. bovigenitalium* was most often identified in vaginal samples, but was never identified from foetal abortion samples. In contrast, *U. diversum* was found in both vaginal swab and abortion samples, as were *M. bovis, M. bovirhinis* and the ubiquitous *M. arginini* (Table 3). *M. bovis* was also the most frequent mycoplasma identified from joints (52%), although *M. alkalescens* (21%), *M. bovirhinis* (11%)*, M. arginini* (0.7%), *M. canadense* (0.4%), and *M. canis* (0.2%) were also found in joints, albeit less frequently than *M. bovis* (Table 3). *M. bovoculi* was only identified in bovine eye samples. In cases with more unusual clinical presentations, *M. bovis* was identified in the brains of two calves displaying neurological signs including head tilt, mild ataxia and reduced menace response. In both cases histopathological examination of the brain identified lesions consistent with *M. bovis* infection, and as one animal was considered unusually old at 12 months of age, the presence of *M. bovis* was confirmed with specific immunohistochemistry labelling. In a third case, involving a pre-weaned calf displaying similar symptoms following treatment for pneumonia, *M. bovis* was identified in cerebrospinal fluid. Histopathology in this case identified a fibrinosuppurative, granulomatous leptomeningitis with the formation of pyogranulomatous nodules/abscesses and, in the absence of positive Gram staining for other bacteria, these findings were suggestive of *Mycoplasma* spp. infection. The combined histopathological and microbiological findings confirmed *M. bovis* as the primary pathogen in these cases. *M. canis* was identified in a case of calf meningitis in 2018, but histopathology identified Gram-positive cocci associated with the brain lesions indicating the mycoplasma was not clinically significant in this case. *M. bovirhinis* was identified from a brain swab and lung of a pneumonic calf in a different case, but death was attributed to *Histophilus somni*. *M. bovis* (*n* = 1) was identified from the pericardium of a case of *M. bovis* pneumonia with accompanying arthritis, while *M. dispar* was identified in the same tissue from a case of bovine respiratory disease associated with *Mannheimia haemolytica*. *M. californicum* (lung), *M. equirhinis* (nasal passage),* Ca.* M. haemobos (blood), *M. fermentans* (lung), *A. equifetale* (lung), and *A. oculi* (foetal abortion) comprised other notable rare and unusual identifications from cattle over the time period (Table 1 and Table 3).

**Table 3:** *Mollicutes* species identified from different anatomical sites in bovine, ovine and caprine species

***Mollicutes*** species Identified^**a**^	Brain	Ear	Eye	Foetal/Abortion Material	Foot	Genital	Joint	Liver	Lung	Lymph Tissue	Milk	Nasal/ Pharyngeal	Pericardium	Pleural fluid	Semen	Small intestine	Spleen	Blood	Udder
*A. equifetale*	0	0	0	0	0	0	0	0	1	0	0	0	0	0	0	0	0	0	0
*A. granularum*	0	0	0	0	0	1	0	0	1	0	0	0	0	0	0	0	0	1	0
*A. laidlawii*	1	1	1	0	1	6	0	0	24	0	0	2	0	0	0	0	0	0	0
*A. oculi*	0	0	0	1	0	0	0	0	0	0	0	0	0	0	0	0	0	0	0
*M. alkalescens*	0	0	7	0	0	7	17	1	488	2	24	30	0	3	0	0	0	0	2
*M. arginini*	1	0	4	5	0	5	6	0	482	2	7	30	0	0	0	1	0	0	0
*M. bovigenitalium*	0	0	0	0	0	30	0	0	15	0	1	0	0	0	0	0	0	0	0
*M. bovirhinis*	2	4	0	2	0	8	9	0	615	2	15	101	1	0	0	0	0	0	1
*M. bovis*	4	7	1	6	0	3	43	0	1000	1	115	93	1	0	0	1	1	1	0
*M. bovoculi*	0	0	10	0	0	0	0	0	0	0	0	0	0	0	0	0	0	0	0
*M. californicum*	0	0	0	0	0	0	0	0	1	0	0	0	0	0	0	0	0	0	0
*M. canis*	1	0	0	0	0	2	2	0	124	0	7	6	0	0	0	0	0	0	0
*M. canadense*	0	0	1	0	0	2	3	0	50	0	10	14	0	0	1	0	0	0	1
*M. conjunctivae*	0	0	174	0	0	0	0	0	0	0	0	0	0	0	0	0	0	0	0
*M. dispar*	0	0	0	1	0	0	0	0	440	0	1	7	1	0	0	0	0	0	0
*M. equirhinis*	0	0	0	0	0	0	0	0	0	0	0	1	0	0	0	0	0	0	0
*M. fermentans*	0	0	0	0	0	1	0	0	1	0	0	0	0	0	0	0	0	0	0
*Ca.* M. haemobos	0	0	0	0	0	0	0	0	0	0	0	0	0	0	0	0	0	8	0
*M. ovipneumoniae*	0	1	1	0	0	1	2	0	449	2	2	72	1	0	0	0	0	0	0
*M. ovis*	0	0	0	0	0	0	0	0	0	0	0	0	0	0	0	0	0	1	0
*M. wenyonii*	0	0	0	0	0	0	0	0	0	0	0	0	0	0	0	0	0	177	0
*U. diversum*	0	0	0	6	0	8	0	0	112	0	1	0	0	0	0	0	0	0	0
**Total**	**9**	**13**	**199**	**21**	**1**	**74**	**82**	**1**	**3803**	**9**	**183**	**356**	**4**	**3**	**1**	**2**	**1**	**188**	**4**

^**a**^Due to unspecified sampling site, 623 identifications are not displayed

In small ruminants, *M. ovipneumoniae* (54% of identifications), *M. arginini* (29%) and *M. conjunctivae* (17%) were most prevalent (Table 2)*. M. ovipneumoniae* was predominately identified from the lungs (*n* = 449 (73%) identifications) and upper respiratory tract (*n* = 72 (12%)), with the remainder from ear, eye, genital, joint, lymph, milk and pericardial samples (Table 3). *M. conjunctivae* was detected only from eyes. Milk samples from mastitic sheep and goats infrequently yielded *Mycoplasma* sp.; with only two identifications of *M. ovipneumoniae* and six of *M. arginini* over the fourteen year period. The haemoplasma *M. ovis* was identified just once, in 2017, from a sheep with a reported blood packed cell volume of 12%, yet the animal appeared well. *M. bovis* was identified twice in lambs; in one case from lung tissue, and the second from a brain swab from which *M. arginini* was also found as a co-infecting organism (Table 2 & Table 3).

The ubiquitously occurring *M. arginini* was identified from a number of body sites in bovine, ovine and caprine hosts, mostly as a co-infecting *Mycoplasma* species in lung and nasal/pharyngeal samples, but also, less frequently in brain, eye, foetal material, genitals, joint, lymph tissue, milk and small intestine (Table 3).

Identification of two or more *Mycoplasma* species in a given sample was not uncommon. *M. bovis* and *M. alkalescens* most commonly co-existed in an average of 11 samples annually (range of 3 to 17). Less common were *M. bovis* and *M. arginini* (6 occurrences per year, range of 0 to 21), *M. bovis* and *M. bovirhinis* (6 occurrences per year, range of 1 to 16), and *M. dispar* and *M. bovirhinis* (5 occurrences per year, range of 0 to 19); other mixed infections of note were *M. bovis* and *M. dispar* which were more common from 2011 onwards (ranged from0 to 11 annually), and *M. bovis* and *M. canadense* (ranged from 0 to 10). In ovine/caprine samples *M. ovipneumoniae* accompanied by *M. arginini* was not uncommon, although observations varied considerably, ranging from 3 to 26 annually.

### Recorded diagnoses

**Fig. 2:**
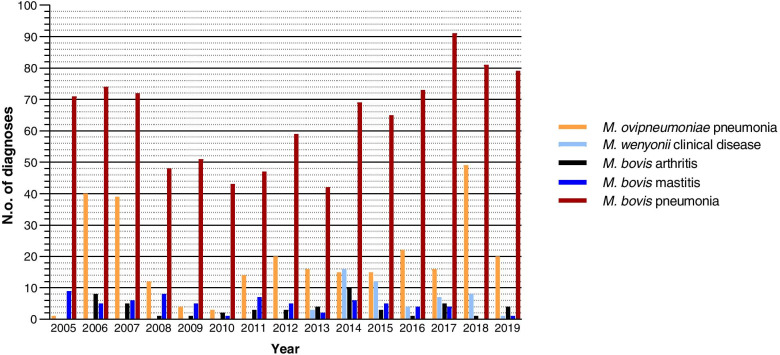
Mycoplasma disease diagnoses made by APHA Veterinary Investigation Centres from 2005 to 2019 in England and Wales using a mixture of observations including clinical signs, post-mortem findings, diagnostic tests and histology

The mycoplasma diagnoses recorded by APHA VICs, and collated from the VIDA database, included *M. bovis* arthritis, mastitis and pneumonia, *M. wenyonii* clinical disease and *M. ovipneumoniae* pneumonia. In England and Wales, between 2005 to 2019 *M. bovis* pneumonia was diagnosed on 965 occasions, ranging from 42 to 91 diagnoses annually (Fig. 2). From 2007 there was a general decline in pneumonia diagnoses, but since 2011 incidence has returned to, or exceeded, that of the early years of the study. In comparison *M. bovis* arthritis and mastitis diagnoses form only a small proportion of the total involving this pathogen, 51 and 68 diagnoses, respectively, between 2005 and 2019. *M. bovis* arthritis diagnoses generally remained low. *M. wenyonii* clinical disease diagnoses in England and Wales were first recorded in 2013, with 51 diagnoses made by the end of 2019. Although an initial increase in incidence was observed, diagnoses have subsequently decreased (Fig. 2).

Two hundred and eighty-six *M. ovipneumoniae* pneumonia diagnoses were recorded by APHA VICs in England and Wales over the reporting period, but annually the number of diagnoses ranged considerably, between 1 and 49 (Fig. 2).

## Discussion

Despite twenty-two different *Mollicutes* species identified in ruminant samples from English and Welsh farms submitted to APHA for diagnosis over the 14 year period, *M. bovis* and *M. ovipneumoniae* were the most commonly occurring mycoplasmas from cattle and small ruminants, respectively. This finding is consistent with the predominance of these *Mycoplasma* spp. reported between 1990 and 2000 [8].

In keeping with a previous report by Ridley and Hateley [11] based on assessment of VIDA data, *M. bovis* was primarily associated with cases of calf pneumonia of which, on average, 64% comprised pneumonia cases submitted between October and the end of March each year. Ridley and Hateley [11] included data from SRUC Veterinary Services Disease Surveillance Centres in Scotland, whereas in the present study, cases and data generated by SRUC were not included and may have resulted in the exclusion of some diagnoses from cases originating from farms in England. It should be noted that the results presented cannot be considered complete and comprehensive data on the *Mycoplasma* species present in diseased ruminants in England and Wales between 2005 and 2019, because the decision to look for mycoplasmas was made post-mortem or in the field by veterinarians, not all of whom would have initially considered mycoplasmas as the most likely cause of disease. France, who since 2003 have also operated an extensive surveillance network for mycoplasmoses of ruminants, (VIGIMYC), have also reported a high incidence of *M. bovis*-associated bronchopneumonia principally involving young animals [12, 13]. *M. bovis* is a recognised highly prevalent and significant respiratory pathogen of young cattle in many other countries worldwide and especially in North-Western Europe [14–17]. However, *M. bovis* pneumonia was also seen in older cattle in the present study, with 13% of diagnoses occurring in those aged over 1 year, in-line with a 2–16% range reported for Great Britain [11]. *M. bovis* affecting older animals has previously been described in Northern Italy [18], where it appears to circulate in fattening farms via mixing of new incoming bulls with those already present on the unit [19]. Although *M. bovis* can be a sole cause of respiratory disease, accounting for 28% of the pneumonia diagnoses included in the present study, it can also form part of the multifactorial bovine respiratory disease complex (BRDC) [20]. Diagnoses mined from VIDA also included viral agents such as bovine respiratory syncytial virus, parainfluenza virus type 3, bovine viral diarrhoea virus, bovine herpesvirus-1, and bacterial pathogens *Trueperella pyogenes*, *M. haemolytica*, *Pasteurella multocida*, *Histophilus somni, Salmonella* Dublin and *Bibersteinia trehalosi*. However, in some cases although *M. bovis* was identified, other disease agents, including those above, may have been considered of greater clinical significance in observed disease.

In contrast to pneumonia, which accounted for 89% of *M. bovis* diagnoses, the number of *M. bovis*-associated cases of mastitis (6%) and arthritis (5%) submitted to APHA generally appeared low in England and Wales, again in-line with the picture reported for Great Britain in 2018 [11]. This differs to the USA where *M. bovis* infection mainly manifests as clinical mastitis causing major economic and welfare concerns [21]. It should be noted that mastitis screening including *M. bovis* is also offered by private laboratories, and as such the proportion of cases submitted to APHA, and included in VIDA, may be under-represented in the present data-set. Other pathogens were also diagnosed as mastitis agents alongside infection with *M. bovis*; 71% of *M. bovis*-associated mastitis diagnoses involved just *M. bovis*, with the remaining also associated with *Escherichia coli*, *Streptococcus uberis*, *Trueperella pyogenes*, *Bacillus* spp. or *Staphylococcus* spp. In Switzerland, while *M. bovis* has historically been associated with pneumonia, since 2007 increases in mastitis outbreaks have been reported [22, 23], while in Finland, a country free of *M. bovis* until 2012, *M. bovis*-infected semen introduced into naïve herds resulted in pneumonia, mastitis and arthritis [24]. New Zealand identified *M. bovis* for the first time in 2017 in dairy herds and made the bold decision to eradicate this organism to prevent future losses due to the disease, with the eradication programme ongoing [4] and the definitive source of introduction has not to date been identified. Once established in a herd the organism is difficult to eradicate, therefore early antimicrobial treatment combined with separation of affected animals is important to limit spread. There is still no efficient commercial vaccine available against *M. bovis* in Europe and several studies have highlighted reducing susceptibility of *M. bovis* to many of the antimicrobial families currently used. Therefore autogenous vaccines remain in use for disease control. A bacterin vaccine produced in the United States, Myco-B ONE DOSE™ (American Animal Health), which comprises three *M. bovis* strains is also now currently being trialled by some farms in Great Britain under special licence from the Veterinary Medicines Directorate as an alternative for control of *M. bovis*. Systemic infection manifesting as more than one clinical syndrome presenting concurrently, such as pneumonia and arthritis has been previously reported, with concurrent syndrome diagnoses observed in this study [14, 25–28]. Individual strains have been recovered from different sites within a host, and genetic similarity evaluations by pulsed-field gel electrophoresis, multi-locus sequence typing and most recently, whole genome sequencing, have all demonstrated *M. bovis* strains from different farms and countries that are associated with cases of pneumonia, mastitis and arthritis are often indistinguishable [29–33]. These findings would suggest that particular disease syndromes may be less associated with pathogen factors and more to do with host and environmental factors [34], although the mechanisms by which the organism is able to evade the immune system to circulate in the host remains an important knowledge gap [35].

*M. bovis* has previously been isolated from the brains of calves in Great Britain, including one with neurological signs [36]. In the present study, *M. bovis* was the primary pathogen identified in three cases of neurological disease, one of which was in an animal 12 months of age. The mechanism by which this particular tissue tropism occurs has not been fully elucidated. However, it is postulated that *M. bovis* infection by colonisation of the inner ear and subsequent damage may lead to direct entry to the brain, or, following development of *M. bovis* pneumonia, the pathogen may enter the brain in ascending infection via the Eustachian tubes or, where otitis is not evident, via the blood stream [33, 36]. Together with identification in pericardial tissue in the present study and as reported previously [36], these observations serve to further highlight the apparent invasive properties of this pathogen.

Although *M. bovirhinis* was again the second most common species in the present period, *A. laidlawii* was rarely identified, most likely due to the PCR-DGGE approach reducing the likelihood of mycoplasmas present in samples being overwhelmed by this faster growing mollicute. In contrast, *M. alkalescens* and *M. dispar* notably rose in occurrence, particularly in the more commonly submitted respiratory sample types. *M. dispar,* was rarely identified between 1990 and 2000 [8], but is considered a pathogen of cattle due to its association with respiratory disease in calves and its ability to elicit pneumonia in experimental infections and cytopathic effects on cultured bovine cells [37–39]. However, the organism is also present in the respiratory tract microbiome of apparently healthy animals, as well as those with respiratory disease [40, 41]. In the present study *M. dispar* was overwhelmingly identified in lung tissue samples (98%) collected from post-mortem examinations with gross evidence of pneumonia. With disease pathogenesis associated with this organism in the lungs now well documented, this finding of *M. dispar* as a predominant mycoplasma in lower respiratory tract lesions further supports its role as a pathogen of respiratory disease. Given its fastidious cultural requirements, the true prevalence of *M. dispar* is likely to be underestimated. Moreover, despite improved awareness of *M. dispar* as a respiratory pathogen in recent years, not all cases of respiratory disease will be investigated for the involvement of mycoplasmas.

The 16S rDNA-based identification through PCR-DGGE has reduced labour-intensity associated with isolation of mycoplasma-like organisms, followed by biochemical, serological and specific PCR tests on DNA prepared from isolated colonies to identify species. PCR-DGGE identifies multiple species, including those belonging to the genera *Mycoplasma, Ureaplasma* and *Acholeplasma*, irrespective of successful culture. Moreover, DNA of non-cultivable mollicutes, such as the haemoplasmas can be directly detected from submitted EDTA blood samples.

Several mixed infections, which often involved *M. bovis* in cattle samples, were recorded using PCR-DGGE over the years. *M. bovis* and *M. alkalescens*, and *M. bovis* with *M. arginini* or *M. bovirhinis*, and *M. dispar* with *M. bovirhinis* were most common in cattle. In such cases *M. bovis* and *M. dispar* are considered the primary mycoplasma pathogens with possible opportunistic exacerbation by the other *Mycoplasma* species [5]. As previously indicated, given the multifactorial nature of BRDC *Mycoplasma* species can act opportunistically to exacerbate disease when other viral and bacterial agents are present. In France, *M. bovis* with *M. bovirhinis* or *M. arginini* are most commonly found together as mixed infections [13].

*M. bovigenitalium* was most common in genital tract samples and such infections have been associated with inflammatory syndromes and fertility problems in cattle previously [42–44]. Although it grows readily in culture, *M. bovigenitalium* is not always considered in the differential diagnosis of reproductive disease. This may also be true for *U. diversum* which is associated with infertility and abortion cases, with in vitro evidence supporting invasion of bovine spermatozoids [45, 46], but again lack of awareness of *Ureaplasma* spp. as a potential aetiological agent, and possibly costs associated with testing, may mean submission of samples and subsequent diagnosis is infrequent. *U. diversum* was not identified between 2005 and 2010, following which, it was identified mainly in lung tissue, and seldom from genital and foetal/abortion samples. Its role in respiratory disease is, however, generally considered opportunistic, rather than as a primary cause of disease [47]. *M. bovigenitalium* was also found in lung samples, most often in younger animals (below 5 months), and once in milk; involvement of this organism in mastitis has been documented but there is no evidence to date for a role in respiratory disease [48]. The identification of *M. alkalescens* and *M. canadense* from milk of mastitic cattle is unsurprising and is in keeping with previous reports as agents involved in mastitis, either as primary pathogens or in association with species like *M. bovis* and other bacteria [16, 21, 49]. *M. californicum,* previously associated with mastitis outbreaks in a number of countries [21, 50–52], was only identified on a single occasion from a pneumonic lung, where *P. multocida* and *B. trehalosi* were considered the primary pathogens.

Identification of haemoplasmas is only achieved via blood smears and molecular techniques with the sensitivity of PCR considered superior [53]. Although both *M. wenyonii* and Ca. M. haemobos were identified in cattle, *M. wenyonii* was more prevalent in England and Wales. Co-identification of *M. wenyonii* and Ca. M. haemobos was only observed once. Both are associated with clinical anaemia, but *M. wenyonii* has been associated with pyrexia, hindlimb and/or udder oedema and prefemoral lymphadenopathy [6, 54, 55]. However, owing to inconsistent observations pertaining to infection and clinical disease, the clinical relevance of these organisms is still debated [56, 57]. Nonetheless, anecdotally, these organisms appear to circulate intermittently in the blood and may remain undetected in blood samples by PCR, despite apparent clinical signs. *M. ovis* was identified only once, from an anaemic sheep. While low prevalence of this haemoplasma may be suggested, intermittent circulation of the organism, challenging its detection, and low-profile association with anaemia in sheep may result in underestimation of occurrence. Transmission of the haemoplasmas is thought to be through contact with blood and body fluids and associated with biting insects. Affected animals often recover without treatment, but carrier-status is unknown [6].

Further atypical findings included *M. fermentans* from a bovine lung sample in a case of possible embolic pneumonia where udder cleft dermatitis was also present, and the penis of a sheep. This organism has been previously identified from genitals of sheep, including a vaginal ulcer and ulcerated penis [8], but evidence of *M. fermentans’* involvement in digital dermatitis in cattle remains inconclusive [58–60]. The sole identification of *A. oculi* in connection with a case of bovine abortion was not considered clinically significant despite its previous association with infectious keratoconjunctivitis and genital lesions in goats and sheep [61–63]; any role as a potential pathogen remains unclear.

In sheep and goats *M. ovipneumoniae* was the most frequently identified *Mycoplasma*, confirming its importance as a respiratory pathogen in these hosts in England and Wales. The number of identifications has increased since the last examination period of 1990–2000 [8]. This organism is implicated in chronic non-progressive pneumonia of low mortality, but varying morbidity, affecting young and adult animals, sometimes referred to as ‘coughing syndrome’, with more severe pneumonia and ill-thrift presenting in younger animals [5, 64, 65]. Subclinical infection of animals with *M. ovipneumoniae* is also reported, and this mycoplasma is thought capable of initiating pneumonia under certain conditions, predisposing the animals to further infection with secondary bacteria such as *M. haemolytica,* progressing to severe pneumonia [5]. *M. ovipneumoniae* was often co-identified with *M. arginini* in the present study, but while the latter organism is not considered to play a significant role in respiratory disease in small ruminants [66] it may contribute to disease severity. Recorded *M. ovipneumoniae* pneumonia diagnoses did not always correspond with *M. ovipneumoniae* identifications as observed in years 2005–2007, 2009, 2010 and 2015. This is due to differences in the number diagnoses held in the VIDA database and the number of mycoplasma identifications held separately by APHA Mycoplasma Team. This may be attributed to; a small number of cases being directly submitted to the Mycoplasma team, linked submissions in a single outbreak of disease, or other agents of disease being considered more significant, such as *Mannheimia* spp. and *B. trehalosi*, in diagnosis. The population structure of *M. ovipneumoniae* isolates in France was recently investigated following an apparent increase in prevalence since a report by Chazel et al.*,* [12], demonstrating increasing recognition and interest in this organism as an important pathogen of small ruminants in Europe [67]. As with *M. bovis* the organism can be difficult to control once established and with no commercial vaccines yet available, early antimicrobial therapy is usually advised.

Identification of *M. bovis* from two cases involving lambs, was not considered clinically significant as other infectious agents were found. A role of *M. bovis* in clinical disease of sheep and goats cannot be excluded, despite being rare, as there is experimental evidence supporting induction of mastitis in ewes [68], but it is not often considered in small ruminant diagnosis. Infection of these hosts with *M. bovis* most likely occurs following mixing or co-inhabitation with cattle, and interestingly, at one of the case premises both cattle and sheep were registered, but the other premises had only registered sheep. *M. conjunctivae*, a major cause of IKC was identified in 17% of small ruminant samples, higher than that previously reported by Ayling, Bashiruddin [8] (10%). The organism can invade ocular structures and can cause severe clinical signs that can hamper vision and lead to the perforation of the cornea [69]. *M. conjunctivae* has been identified in flying insects found around affected sheep and wild caprinae hosts [70], suggesting a potential vector transmission route [71]. *M. bovigenitalium*, formerly ovine group 11 and previously implicated in cases of infertility in ewes between 1998 and 2000 [8], was not identified during this study.

Other than milk from a single goat in a consignment of four imported into Wales in 2014 positive for *M. agalactiae* (Anon, 2014), no other contagious agalactia causing *Mycoplasma* species were identified in this study, suggesting that the disease continues to be absent in GB, despite the increase in herds of milking sheep and goats in recent years [72]. APHA continues to conduct passive microbiological surveillance of mastitis cases in small ruminants, and other pertinent cases of clinical disease, as well as post-import serological testing to detect previous exposure to *M. agalactiae*.

## Conclusion

A variety of *Mollicutes* species were identified in samples received as diagnostic submissions originating from ruminant livestock farms in England and Wales by the Mycoplasma Team at APHA. Not all species identified have known roles in clinical disease. *M. bovis* and *M. ovipneumoniae* were the most frequent species identified from samples of bovine and small ruminant origin, respectively. *M. ovipneumoniae* appears to be rising in prominence in the differential diagnoses of pneumonia cases in sheep and goats, while *M. conjunctivae* continues to be implicated in IKC. The continued high prevalence of *M. bovis,* particularly in association with bovine respiratory disease highlights challenges faced by farming associated with continued lack of effective commercial vaccines, effective treatments, and associated transmission prevention measures.

## Materials & methods

### Sample collection and culture conditions

Until 2007 mycoplasma-like organisms were cultured from post-mortem tissue or fluids, or, clinical swabs, EDTA anticoagulated blood samples, joint fluid and tracheobronchial lavages from live animals at APHA regional laboratories, at the request of the veterinary investigation officers who suspected mycoplasma infection based on the clinical history and gross post-mortem findings, including pneumonia, mastitis, arthritis, reproductive or eye disease. In such cases, samples were processed by a standardised procedure which included culture at 37 °C in Eaton’s medium with serial dilution to enrich for *Mollicutes*, followed by sub-culture onto agar plates at 37 °C in a 5% CO_2_ atmosphere to confirm the presence of mycoplasma-like organisms [73]. Any suspect organisms were submitted to the Mycoplasma Team at APHA Weybridge for species identification. Since 2007, samples without prior cultural enrichment have been submitted from regional laboratories, the APHA Veterinary Investigation Centres (VICs), and since 2014, this also included partner providers of post-mortem and pathology services, and direct submissions from veterinary practices and private diagnostic laboratories. On receipt by the Mycoplasma Team at Weybridge, samples were inoculated into Eaton’s broth, with tissues first macerated in broth, then serially diluted in Eaton’s to reduce effects of tissue growth inhibitors and contaminating bacteria [73].

### Molecular identification

Following dilution in culture broth a 1 mL aliquot was removed and stored at 4 °C (+/− 1.5 °C), with a second aliquot taken following a 24 h enrichment period and pooled with the first. This pool was pre-digested with proteinase K (400 μg) (Qiagen, Manchester, UK) in a sodium dodecyl sulphate tissue lysis buffer (Qiagen) at 55 °C for 3 hours, prior to automated DNA preparation using a Maxwell® 16 System with the Maxwell® 16 Blood DNA Purification Kit (Promega, Hampshire, UK). DNA was prepared from blood samples (100 μL) using the DNeasy Blood & Tissue Kit (Qiagen) according to the manufacturer instructions.

A conventional endpoint PCR was used to amplify the V3 region of the 16S rDNA from 1 μL of extracted DNA, and PCR amplicons were analysed by DGGE using INGENY phorU 232 apparatus (GRI Molecular Biology) and Cleaver Scientific VS20WAVE-DGGE (Warwickshire, UK) electrophoresis systems, as previously described [9, 10] with the following modification; high and low denaturing solutions were adjusted to 68 and 15%, respectively, for use with VS20WAVE-DGGE system. Samples submitted for detection of haemoplasmas were tested with primers and PCR conditions described by McAuliffe, Ellis [9], with all other *Mollicutes* species identified with primers and conditions described by McAuliffe, Ellis [10]. Species identification was determined by interpretation of denaturing profiles compared to standard positive controls. Where no identification was made from the initial test, but cultural characteristics indicated mycoplasma-like growth, additional DNA preparations from growing cultures and PCR-DGGE analyses were undertaken. Specific PCRs for *M. conjunctivae* [74], *M. agalactiae* [75], *M. ovipneumoniae* [65], *M. bovis* [75, 76], and *M. canis* (K. Johansson and R. D. Ayling, unpublished observations), or sequencing of PCR amplicons were performed to support species identification where required.

### Case and diagnoses data

Case data was collated from veterinary diagnostic submissions to the Great Britain veterinary surveillance network, for England and Wales. This comprises APHA Veterinary Investigation Centres (VICs) and partner providers of post-mortem and pathology services. Accompanying diagnosis data, where available, was retrieved from the Veterinary Investigation Diagnosis Analysis (VIDA) database. For a small number of cases, samples were submitted directly to APHA Weybridge for mycoplasma examination, with limited clinical data.

## Data Availability

All data generated or analysed during this study are included in this published article.

## Abbreviations

APHA: Animal and Plant Health Agency
Defra: Department for Environment, Food and Rural Affairs
DGGE: Denaturing gradient gel electrophoresis
VIDA: Veterinary Investigation Diagnosis Analysis
VIC: Veterinary Investigation Centre
